# Pan-cancer analysis of the role of MPP7 in human tumors

**DOI:** 10.1016/j.heliyon.2024.e36148

**Published:** 2024-08-10

**Authors:** Xiaotong Xu, Weyland Cheng, Shuai Zhao, Yuchun Liu, Lifeng Li, Xiaorui Song, Yaodong Zhang, Cong Ding

**Affiliations:** Children's Hospital Affiliated to Zhengzhou University, 33 Longhu Waihuan East Road, Zhengzhou, Henan, 450018, PR China

**Keywords:** MPP7, Cancer genetics, Epigenetics

## Abstract

MAGUK p55 subfamily member 7, a part of the membrane palmitoylated protein subfamily, is an essential adapter that promotes epithelial cell polarity and has increasing significance in multiple cancers, including esophageal cancer, clear cell renal cell carcinoma, breast cancer, and pancreatic ductal adenocarcinoma. This paper aims to determine the effect of the MAGUK p55 subfamily member 7 in various tumor types using The Cancer Genome Atlas and Genotype-Tissue Expression database. A variety of software and web platforms, such as cBioPortal, GEPIA2, TIMER2, UALCAN, R, STRING, and DAVID, were used to obtain and analyze data. Notably, low expression of MAGUK p55 subfamily member 7 was observed in most cancers. In addition, low expression of MAGUK p55 subfamily member 7 predicted poor prognoses in cancer patients. Mutation was the most frequent genetic alteration type in MAGUK p55 subfamily member 7, with the phosphorylation sites identified as S412 and S490 in various cancers. Furthermore, expression of MAGUK p55 subfamily member 7 was associated with cancer-related fibroblasts and CD8^+^ T cells. Gene enrichment analysis indicated that MAGUK p55 subfamily member 7 influences cancer through the Rap1 signaling pathway. This paper elucidates the biological significance of MAGUK p55 subfamily member 7 in human pan-cancer prognosis and immune response.

## Highlights

• MPP7 is a potential biomarker for various cancers.
• MPP7 under expression is relevant to a poor prognosis.
• MPP7 expression is related to tumor immune cell infiltration.
• MPP7 is involved in tumorigeesis through the Rap1 signaling pathway.

## 1 Introduction

Cancer is an important cause of death and has a significance influence on global human health as well as economic stability [1]. Although the treatment effect of cancer has improved, the prognosis and survival probability for cancer patients remain poor due to multiple factors such as adverse side effects [2] and drug resistance. Consequently, the discovery of novel pan-cancer biomarkers and therapeutic targets is essential to improving public health.

Cell polarity is closely associated with tumorigenesis and tumor progression [3]. Abnormal localization and expression of polar proteins can significantly impair cell morphology and cell–cell adhesion, promoting the onset, invasion, and metastasis of tumors [4]. Cell adhesion, junction, and polarity are regulated by MAGUK p55 subfamily member 7 (MPP7), a member of the membrane palmitoylated membrane protein subfamily. Some researchers have explored the function of MPP7 in tumorigenesis. In breast cancer, ectopic expression of MPP7 promotes cell migration and invasion by modulating epithelial–mesenchymal transition and activating the epidermal growth factor receptor signaling [5]. MPP7 has been characterized as a biomarker for esophageal cancer [6]. Notably, immunohistochemical results demonstrated that high levels of MPP7 were related to poor prognosis in patients with esophageal cancer. Furthermore, MPP7 has been implicated in the regulation of autophagy in pancreatic ductal adenocarcinoma (PDAC) [7]. In this study, MPP7 directly interacted with Yes-associated protein 1 to control hypoxia-induced autophagy and eventually decreased PDAC cell survival. Collectively, these studies indicate that MPP7 is involved in the tumorigenesis of different cancers. Nevertheless, further investigation is warranted to fully understand the pan-cancer function of MPP7 in tumorigenesis and its potential as a therapeutic target.

The rapid development of biological databases has significantly enhanced the reliability and representativeness of bioinformatic analyses using extensive datasets and sophisticated computational methods. In this research, we used bioinformatics methods to investigate the expression profile, functional, genetic changes, immune infiltration, protein phosphorylation levels, prognostic value, and functional enrichment of MPP7 in multiple cancers. This comprehensive assessment underscores the significance of MPP7 in the prognosis of multiple cancers, its potential involvement in several less-studied cancers, the intrinsic molecular mechanisms of MPP7 in human cancer pathogenesis, and the significance of MPP7 in anti-tumor immune responses.

## 2 Methods

### 2.1 Gene expression analysis

Differences in MPP7 expression between tumor cells from diverse types of cancer or specific subtypes and neighboring healthy tissues were evaluated in the Cancer Genome Atlas (TCGA) dataset [8]. Using the TIMER2 website (http://timer.cistrome.org/) "Gene DE" module, MPP7 was queried to determine the expression differences between tumor tissue and adjacent healthy tissues. The box diagrams of differential expression between specific tumor tissues and their corresponding healthy tissue counterparts in the Genotype-Tissue Expression (GTEx) database (log2FC cutoff = 1, *P*-value cutoff = 0.01, “GTEx data, Match TCGA normal”) were generated using the “Expression Analysis Box Plots” module of the GEPIA2 web (http://gepia2.cancer-pku.cn/#analysis) [9]. Additionally, a violin map was generated using “GEPIA2's Pathological Stage Plot” module to show the expression of MPP7 in malignant staging across all tumors. Prior to generating the box or violin plots, the expression data is transformed using the log2 (TPM + 1) method. DepMap was used to analyze MPP7 dependence in different cancer contexts. The Clinical Proteomics Analysis Consortium (CPTAC) datasets in UALCAN (http://ualcan.path.uab.edu/analysis-prot.html) were used to assess protein expression. UALCAN is an online platform for analyzing OMICS cancer data [10]. The protein expression levels of the phosphorylated MPP7 form (at sites S313, S409, S412, and S490) in primary tumors and healthy tissues were compared with reference sequence NP 775767.2. Datasets for breast cancer, head and neck squamous cell carcinoma (HNSC), and lung adenocarcinoma (LUAD) were selected for the comparative analyses.

### 2.2 Survival prognosis analysis

Kaplan–Meier plots depicting overall survival (OS) and disease-free survival (DFS) of MPP7 in all tumors were generated using the GEPIA2's "Survival map" module [9]. Median expression levels were utilized as the threshold to delineate the high expression group from the low expression group. The log-rank test and “Survival Analysis” module of GEPIA2 were used to validate the hypothesis and generate the survival tendencies, respectively.

### 2.3 Genetic alteration analysis

Using the cBioPortal web (https://www.cbioportal.org/), the “TCGA Pan Cancer Atlas Studies” option in the “Quick Select” area was chosen to investigate genomic alterations in MPP7 [11]. We examined the data in the “Cancer Types Summary” module, focusing on changes in the number of copies, and the rate and types of tumor mutation. Furthermore, we visualized the altered sites of MPP7 through graphical illustrations or three-dimensional structural models using the “Mutations” module. We generated Kaplan–Meier plots, along with P-values and log-rank check, to obtain statistics on OS, DFS, and disease- and progression-free disparities in patients with adrenocortical carcinoma (ACC), regardless of the presence of MPP7 genetic mutations.

Tumor mutation burden (TMB) represents the quantity of mutations within a tumor genome, and its value as a marker for identifying patients who may respond well to immune checkpoint inhibitors has been extensively studied. We obtained the somatic mutation information for each patient from the website (https://tcga.xenahubs.net), computed their TMB values, and then used R version 4.0.3 to determine the association between TMB and MPP7. Microsatellite instability (MSI), characterized by extensive length variations in microsatellite sequences due to DNA polymerase slippage, indicates genetic instability and is useful for the detection of cancer. We calculated the MSI scores for patients and subsequently conducted a correlation analysis between the MSI and MPP7 scores using R version 4.0.3.

### 2.4 Immune infiltration analysis

The “Immune-Gene” module of the TIMER2 platform was employed to find the association between MPP7 expression and immune cell infiltration in all tumors. Cancer-related fibroblasts were chosen for further analysis. EPIC, MCPCOUNTER, XCELL, and TIDE algorithms were used to evaluate immune cell infiltration. *P*-values and partial correlation scores were determined using Spearman's rank correlation test with purity correction. The results were graphically depicted as heatmaps and scatter plots.

### 2.5 MPP7-related gene enrichment analysis

The STRING database was searched (https://string-db.org/) [12] for the *Homo sapiens* MPP7 protein. We established the following primary criteria: a low confidence interaction score of 0.150 minimum, network edges indicating “evidence”, and a maximum of 50 interacting partners displayed in the first shell. Notably, only experimentally supported interactions were considered. Next, we retrieved experimentally validated MPP7-binding proteins. We used GEPIA2's "Similar Gene Detection" module to identify the 100 MPP7-associated genes in tumor and healthy tissue datasets. A pairwise Pearson's correlation analysis between MPP7 and these genes was performed using the GEPIA2's "Correlation Analysis" module. The plot displayed the correlation coefficients (R) and *P*-values. We then used the "Gene Corr" module in the TIMER2 program to generate heat maps of the specific genes, including partial correlations and *P*-values from the purity adjusted Spearman's rank correlation approach. Following, we used Jvenn, a tool for building Venn diagrams [13], to compare the genes that bind to MPP7 with the genes that interact with it. We integrated the datasets for enrichment analysis using the Kyoto Encyclopedia of Genes and Genomes (KEGG) and Gene Ontology (GO). Gene records in DAVID were queried using official gene symbols and species-specific criteria to gain functional annotation chart data. We used the R packages “tidyr” and “ggplot2” to create a visual representation of the enriched pathways. The "clusterProfiler" R package was used for GO enrichment analysis. The cnetplot function was used to visualize data on biological processes, cellular components, and molecular functions. R version 4.0.3 was used for the research. *P* < 0.05 was considered statistically significant.

## 3 Results

### 3.1 Pan‐cancer landscape of MPP7 expression

**Fig. 1:**
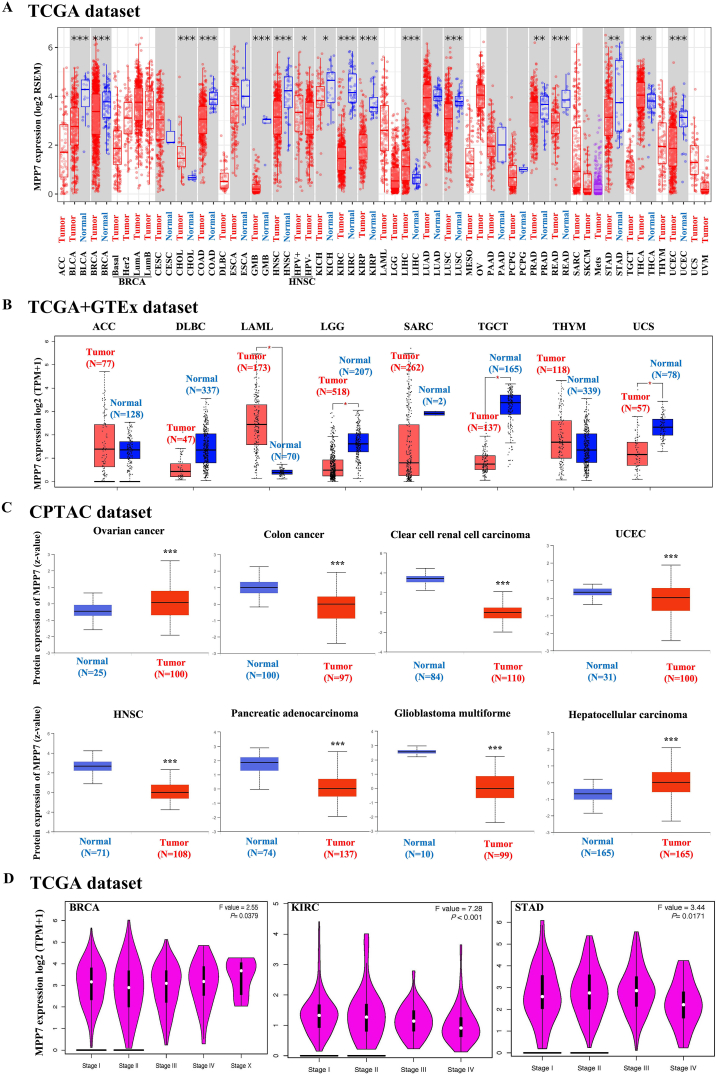
*MPP7* expression in different tumors and pathological stages. A, TIMER2 was used to examine *MPP7* expression in distinct malignancies or subtypes of cancer. *P < 0.05; **P < 0.01; ***P < 0.001. B, For each type of ACC, DLBC, LAML, LGG, SARC, TGCT, UCS, and THYM in the TCGA study, the matching healthy tissues of the GTEx database served as controls. The data for the box plot are presented. *P < 0.05. C, We compared the MPP7 total protein expression between healthy tissues and primary tissues of ovarian cancer, colon cancer, clear cell RCC, UCEC, HNSC, PAAD, and hepatocellular carcinoma using the CPTAC dataset. ***P < 0.001. D, *MPP7* expression by the primary pathogenic stages of BRCA, KIRC, and STAD was examined using TCGA data. For log-scale, Log2 (TPM + 1) was employed. Note: MPP7: MAGUK p55 subfamily member 7, TCGA: The Cancer Genome Atlas, GTEx: Genotype-Tissue Expression project, CPTC: Clinical Proteomic Tumor Analysis Consortium, ACC: Adrenocortical carcinoma, DLBC: Lymphoid neoplasm diffuse large B-cell lymphoma, LAML: Acute myeloid leukemia, LGG: Brain lower grade glioma, SARC: Sarcoma, TGCT: Testicular germ cell tumors, UCS: Uterine carcinosarcoma, THYM: Thymoma, UCEC: Uterine corpus endometrial carcinoma, HNSC: Head and neck squamous cell carcinoma, PAAD: Pancreatic adenocarcinoma, BRCA: Breast invasive carcinoma, KIRC: Kidney renal clear cell carcinoma, STAD: Stomach adenocarcinoma.

The expression level of MPP7 in multiple cancer types in TCGA dataset was analyzed utilizing TIMER2 platform. MPP7 has been detected in breast invasive carcinoma (BRCA), breast invasive carcinoma (BLCA), colorectal adenocarcinoma (COAD), glioblastoma multiforme (GBM), HNSC, kidney renal papillar (KIRP), kidney renal clear cell carcinoma (KIRC), lung squamous cell carcinoma (LUSC), rectum adenocarcinoma (READ), and uterine corpus endometrial carcinoma (UCEC), and was significantly lower compared to matched healthy tissues (*P* < 0.001; Fig. 1A). We analyzed the differential MPP7 expression in uterine carcinosarcoma (UCS), testicular germ cell tumors (TGCT), brain lower-grade glioma (LGG), acute myeloid leukemia (AML), and compared them with healthy tissues in the GTEx dataset for reference (*P* < 0.05; Fig. 1B). The CPTAC data showed that MPP7 total protein expression in primary ovarian cancer, clear cell RCC (ccRCC), colon cancer, GBM, HNSC, UCEC, pancreatic adenocarcinoma (PAAD), and hepatocellular carcinoma (HCC) was significantly lower compared to healthy tissues (*P* < 0.001; Fig. 1C). Moreover, HEPIA2's "Pathological Stage Plot" module was used to determine whether there is a relationship between MPP7 expression and cancer pathological stage, for example, BRCA, KIRC, stomach adenocarcinoma (STAD) (*P* < 0.05; Fig. 1D). Notably, MPP7 expression may be associated with different tumors, including AML, Hodgkin lymphoma, and leiomyosarcoma (Fig. 1D). This analysis aimed to identify significant correlations between MPP7 expression and cancer progression.

### 3.2 Survival analysis data

**Fig. 2:**
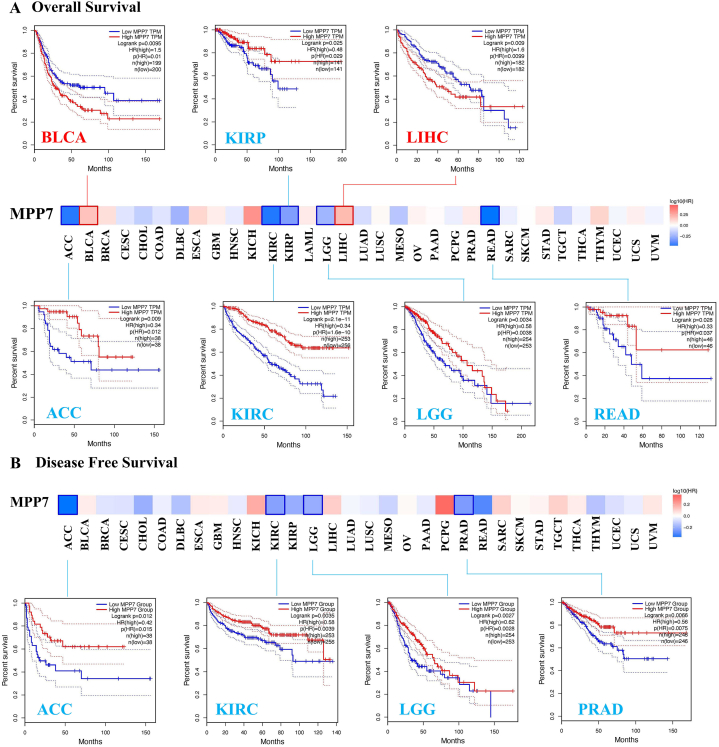
Correlation between *MPP7* expression and survival prognosis of cancers from TCGA data. A, B, The GEPIA2 tool analyzed the overall survival and disease-free survival of multiple tumors in TCGA based on *MPP7* expression. The survival map and positive Kaplan–Meier curves are presented. Note: MPP7: MAGUK p55 subfamily member 7, TCGA: The Cancer Genome Atlas.

Cancer samples were assigned to high-expression groups and low-expression groups according to MPP7 expression. The TCGA dataset was used as a reference to assess the relationship between MPP7 expression and tumor prognosis. Notably, low MPP7 expression was associated with poor OS prognosis for ACC (*P* = 0.012), KIRP (*P* = 0.029), KIRC (*P* = 1.6e-10), LGG (*P* = 0.0036), and READ (*P* = 0.037) (Fig. 2A). DFS evaluation indicated that high MPP7 expression was associated with poor prognosis in ACC (*P* = 0.015), KIRP (*P* = 0.0039), LGG (*P* = 0.0028), prostate adenocarcinoma (PRAD) (*P* = 0.0075) (Fig. 2B). Additionally, elevated MPP7 expression was associated with a poor OS prognosis for both BLCA (*P* = 0.01) and liver hepatocellular carcinoma (LIHC) (*P* = 0.0099) (Fig. 2A).

### 3.3 Genetic change analysis data

**Fig. 3:**
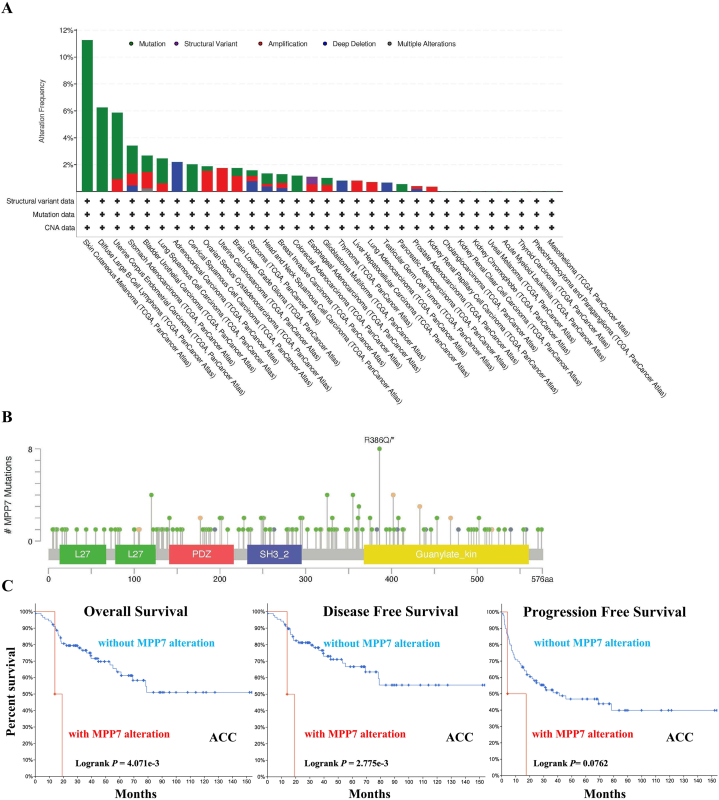
**Mutations in *MPP7* in different tumors from TCGA data.** Mutations in *MPP7* in the TCGA tumors were identified using cBioPortal. A, B, The incidence of mutations based on tumor type and site is illustrated. R386Q is the mutation site with the highest frequency in *MPP7*. C, The probable correlation between mutation status and overall survival, disease-free survival, disease-specific survival, and progression-free survival in patients with ACC using cBioPortal. Note: MPP7: MAGUK p55 subfamily member 7, TCGA: The Cancer Genome Atlas.

**Fig. 4:**
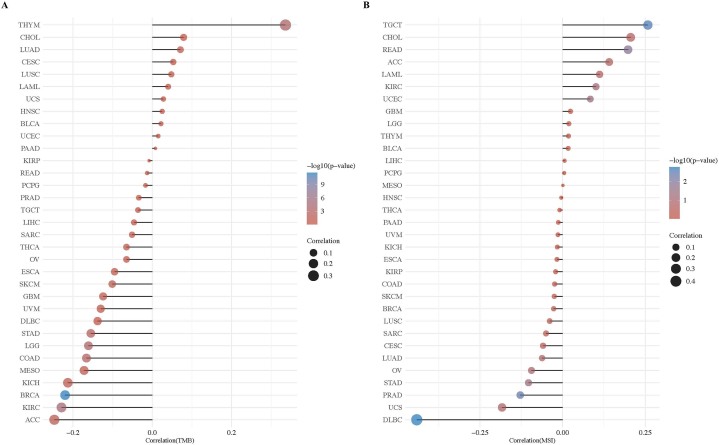
Correlation between *MPP7* expression and cancer mutational burden or microsatellite instability. A, We investigated the possible relationship between *MPP7* expression and TMB using the various cancers from the TCGA. B, We investigated any association between *MPP7* expression and MSI. Note: MPP7: MAGUK p55 subfamily member 7, TCGA: The Cancer Genome Atlas, MSI: Microsatellite instability, TMB: Tumor mutation burden.

Variations in *MPP7* were observed across multiple tumor tissues. Patients with skin cutaneous melanoma (SKCM) exhibited the highest prevalence of *MPP7* alterations (>11 %) (Fig. 3A). Furthermore, copy number alteration type mutations were most prevalent in lymphoid neoplasm diffuse large B-cell lymphoma (DLBC), with a mutation rate of <6 % (Fig. 3A). Moreover, all ACC cases with genetic mutations (approximately 3 %) exhibited a copy number deletion in *MPP7* (Fig. 3A). Fig. 3B presents the types, locations, and frequencies of the genetic changes in *MPP7*. Missense mutations in *MPP7* were the predominant type of genetic alteration, and R386Q alteration in the C-terminal kinase domain was discovered in five, one, and one cases of SKCM, COAD, and UCEC, respectively (Fig. 3B). These mutations could induce a frameshift mutation, changing from arginine to glutamine at the 386th position of the MPP7 protein, subsequently leading to its truncation. We also assessed the association between genetic modifications in *MPP7* and clinical consequences in patients with various cancers. Patients with ACC and MPP7 alterations demonstrated a more favorable prognosis with respect to OS (*P* = 4.071e-3) and DFS (*P* = 2.775e-3), as opposed to progression-free survival (*P* = 0.0762), compared to patients without MPP7 (Fig. 3C). Additionally, we assessed the relationship of MPP7 expression with TMB and MSI in all cancers. The expression of MPP7 was negatively correlated with TMB in various cancers, including ACC, BRCA, colon adenocarcinoma (COAD), kidney chromophobe, malignant mesothelioma, LGG, STAD, DLBC, uveal melanoma, GBM, SKCM, ESCA, ovarian serous cystadenocarcinoma, thyroid carcinoma, sarcoma, LIHC, TGCT, PRAD, pheochromocytoma, paraganglioma, rectal adenocarcinoma, and KIRP (Fig. 4A). A positive correlation was identified in cholangiocarcinoma, cervical squamous cell carcinoma, endocervical adenocarcinoma, thymoma (THYM), LUAD, LUSC, LAML, UCS, HNSC, BLCA, and UCEC. Fig. 4B illustrates that MPP7 expression was negatively related to MSI in DLBC, UCS, PRAD, STAD, ovarian serous cystadenocarcinoma, LUAD, thyroid carcinoma, and positively related to MSI in TGCT, cholangiocarcinoma, rectal adenocarcinoma, ACC, LAML, KIRC, UCEC, GBM, LGG, THYM, BLCA, LIHC, pheochromocytoma, paraganglioma, and malignant mesothelioma.

### 3.4 Protein phosphorylation analysis data

**Fig. 5:**
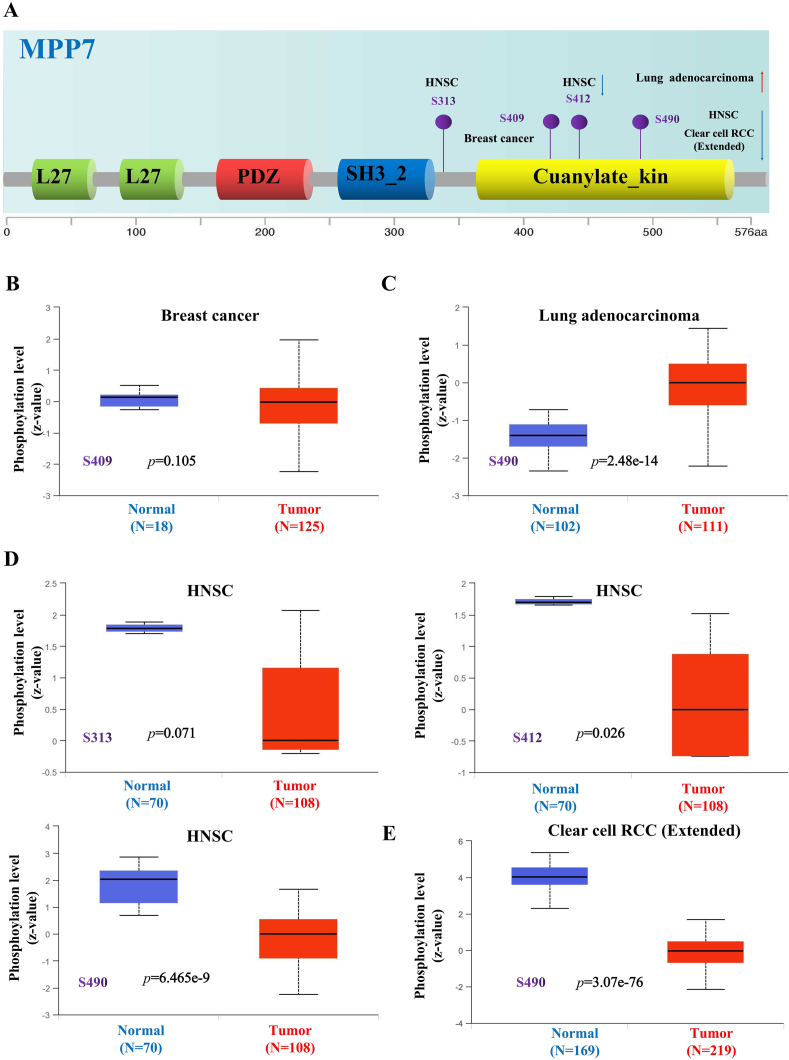
**Phosphorylation analysis of MPP7 protein in different tumors.** The expression pattern of MPP7 phosphoprotein (S313, S409, S412, and S490 sites) was compared between healthy tissue and primary tissue of specified cancers from the CPTAC dataset using UALCAN. A, A graph of the MPP7 protein illustrating the phosphoprotein sites with positive outcomes. B–E, Box graphs for various malignancies are provided, including breast cancer, lung adenocarcinoma, HNSC, and clear cell RCC. Note: MPP7: MAGUK p55 subfamily member 7, HNSC: Head and neck squamous cell carcinoma.

We performed a comparative analysis of the phosphorylated MPP7 protein expression in 11 primary tumor tissues and their normal counterparts using UALCAN. Phosphorylated MPP7 expression was specific to breast cancer, lung adenocarcinoma, HNSC, and ccRCC. Detailed information and significant changes in MPP7 phosphorylation sites are illustrated in Fig. 5A. Increased phosphorylation at S490 within the C-terminal kinase domain was observed in LUAD (*P* = 2.48e-14; Fig. 5C). In contrast, phosphorylation at S412 and S490 within the same domain of MPP7 was decreased in HNSC and ccRCC (all *P* < 0.001; Fig. 5D and E). In breast cancer, phosphorylation at the S409 site showed no significant difference (*P* = 0.105; Fig. 5B).

### 3.5 Immune infiltration analysis data

**Fig. 6:**
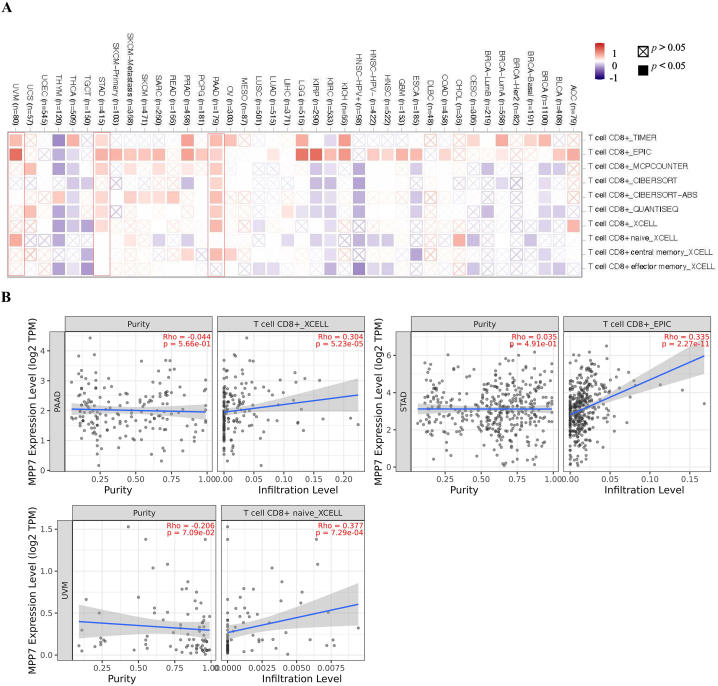
Correlation analysis between MPP7 expression and CD8^+^ T cell immune infiltration. Multiple algorithms were employed to examine the possible correlation between *MPP7* expression and CD8^+^ T cell infiltration rate among various cancers in TCGA. Note: MPP7: MAGUK p55 subfamily member 7, TCGA: The Cancer Genome Atlas.

**Fig. 7:**
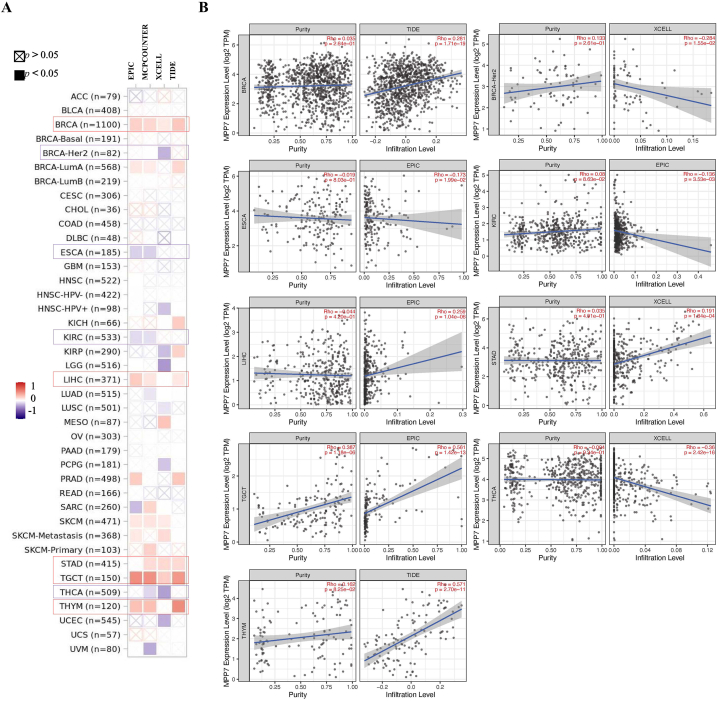
Correlation analysis between MPP7 expression and cancer-associated fibroblast immune infiltration. Numerous algorithms were used to investigate the possible relationships between *MPP7* expression and the cancer-related fibroblast infiltration rate among all cancer types in TCGA. Note: MPP7: MAGUK p55 subfamily member 7, TCGA: The Cancer Genome Atlas.

Tumor-infiltrating immune cells are an important component of the tumor microenvironment, and there is a strong correlation between them and cancer aggressiveness [14]. Cancer-related fibroblasts in the stroma of the tumor microenvironment mediate the role of multiple tumor-infiltrating immune cells [15]. We investigated the connection between immune cell infiltration and *MPP7* expression in various cancer types using algorithms such as CIBERSORT, CIBERSORT-ABS, TIMER, EPIC, QUANTISEQ, TIDE, XCELL, and MCPCOUNTER to analyze data from TCGA. Our analysis revealed a statistically significant positive relationship between the immunological infiltration of CD8^+^ T cells and MPP7 expression in PAAD, STAD, and uveal melanoma, as confirmed by most available algorithms (Fig. 6). Additionally, the MPP7 expression was significantly positively related to the invasion of cancer-related fibroblasts in BRCA, LIHC, STAD, THYM, and TGCT. In contrast, a statistically significant negative relationship was identified for BRCA-Her2, ESCA, KIRC, and thyroid carcinoma (Fig. 7). The resulting scatter plots for these tumors are presented in Fig. 6, Fig. 7. Additionally, the XCELL method confirmed that MPP7 expression was positively related to the immunological infiltration of CD8^+^ T cells in PAAD (Rho = 0.304, *P* = 5.23e-05; Fig. 6).

### 3.6 Enrichment analysis of MPP7-related partners

**Fig. 8:**
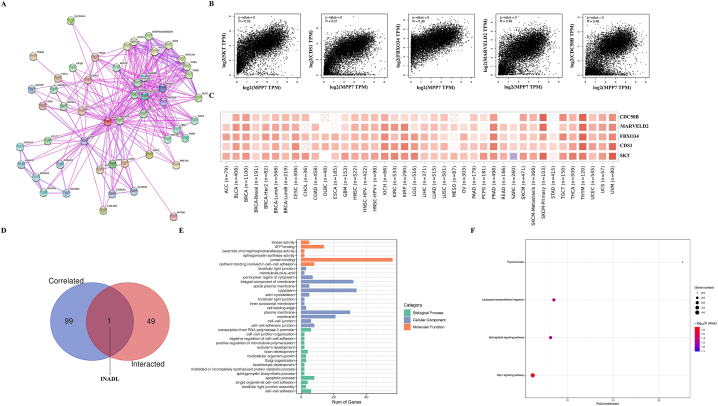
*MPP7*-related gene enrichment analysis. A, MPP7-binding proteins were identified using the STRING program. B, Top 100 *MPP7*-correlated genes in TCGA data were identified using the GEPIA2 approach, and associations between the expression of *MPP7* and selected targeting genes, such as *SKT*, *CDS1*, *FBXO34*, *MARVELD2*, and *CDC50B*, were evaluated. C, The detailed cancer-type-related heatmap data are presented. D. An intersection analysis was performed on the *MPP7*-binding and associated genes. E, The cnetplot for the molecular function records in the GO study. F, KEGG pathway analysis was conducted based on the *MPP7*-binding and interacting genes. Note: MPP7: MAGUK p55 subfamily member 7, TCGA: The Cancer Genome Atlas, SKT: Sickle tail protein homolog, CDS1: CDP-diglyceride synthase 1, FBXO34: F-box-only protein 34, MARVELD2: MARVEL domain-containing protein 2, CDC50B: Cell cycle control protein 50B, GO: Gene Ontology, KEGG: Kyoto Encyclopedia of Genes and Genomes.

We performed multiple pathway enrichment studies of genes and MPP7-binding proteins associated with MPP7 expression to illustrate the molecular mechanisms underlying the function of MPP7 in carcinogenesis. Notably, 50 MPP7-binding proteins were identified using the STRING database, all supported by scientific methods. Fig. 8A displays the network of interactions between these proteins. Furthermore, the GEPIA2 algorithm was used to integrate the TCGA tumor expression data and identify 100 genes related to MPP7 expression. A positive relationship was identified between the expression of MPP7 and sickle tail protein homolog (*SKT*) (R = 0.52), CDP-diglyceride synthase 1 (*CDS1*) (R = 0.51), F-box-only protein 34 (*FBXO34*) (R = 0.49), MARVEL domain-containing protein 2 (*MARVELD2*) (R = 0.49), and cell cycle control protein 50B (*CDC50B*) (R = 0.48) (all *P* < 0.001) (Fig. 8B). A significant proportion of malignancies demonstrated a positive relationship between MPP7 and these five genes, as illustrated in the corresponding heatmap (Fig. 8C). An intersectional analysis of the two teams identified a frequently reported gene, *INADL* (Fig. 8D). The combination of these two datasets enabled enrichment analysis using KEGG and GO. GO analysis illustrated that these genes were predominantly related to mechanisms and cellular biology involving ATP binding, protein binding, integral components of the membrane, cytoplasm, apoptosis, cell–cell adhesion, and other processes (Fig. 8E). Notably, MPP7 may influence the pathophysiology of tumors through the sphingolipid and Rap1 signaling pathways, as revealed by the KEGG analysis (Fig. 8F).

## 4 Discussion

MPP7 plays a major role in regulating polarity, junctions, and cell adhesion [16]. Furthermore, MPP7 regulates the stability of several tumorigenesis-related proteins, thereby affecting cancer progression [[17], [18], [19]]. In the present research, we used a range of bioinformatic tools to reveal abnormal MPP7 expression in human cancers. We then explored the prognostic value of MPP7 expression in different types of cancer and determined the gene mutations and phosphorylation of MPP7. Additionally, we identified the relationship between MPP7 expression and immune cells infiltration levels. Finally, we conducted functional enrichment analysis to elucidate the possible mechanisms by which MPP7 influences cancer pathogenesis. Notably, MPP7 is under expressed and is associated with the growth of multiple cancers. Consistent with previous research, the results from TCGA and GTEx show that MPP7 expression is lower in various cancers than in normal tissues.

Data from the survival prognosis study yielded varied outcomes for various tumors, despite most tumors exhibiting reduced *MPP7* expression. MPP7 promotes breast cancer aggressiveness via epidermal growth factor receptor signaling [5]. We discovered a statistical association between poor OS and high MPP7 expression in patients with breast cancer using the GEPIA2 tool (*P* = 0.01). Furthermore, MPP7 directly binds to Yes-associated protein 1, thereby controlling autophagy in PDAC cells [7]. In contrast, the TCGA study showed no relationship between MPP7 expression and OS prognosis of PDAC. Consequently, large sample sizes are warranted to validate the significance of MPP7 in the developtment of PDAC. Recent studies have shown that *MPP7* is an essential gene associated with ccRCC prognosis, which is aligned to our results [20]. Moreover, our results demonstrate that MPP7 may be a novel biomarker for predicting the outcomes of ACC and LGG.

Various mutations have been identified in *MPP7*. The highest mutation frequency was observed at R386Q in SKCM, COAD, and UCEC. In ACC, *MPP7* mutations were associated with poor survival. Posttranslational modifications of proteins, particularly phosphorylation, modulate protein functions. Notably, reduced MPP7 phosphorylation was observed in HNSC and ccRCC tissues compared with normal tissues, which indicates that MPP7 phosphorylation is crucial for regulating DNA replication during cell division [21]. Nevertheless, the possibility that low MPP7 phosphorylation is an insignificant result of signal deregulation in tumor cells cannot be eliminated.

The tumor immune microenvironment is a critical component of the tumor microenvironment, composed of immune cells that play an essential role in tumors development [22]. Subsequently, confirming new targets for immunotherapy is crucial for improving clinical outcomes; however, the influence of MPP7 on the tumor immune microenvironment has rarely been explored. Infiltrating immune cells are associated with invasion, metastasis, and tumor growth [23]. For instance, cancer-associated fibroblasts can increase tumor development by secreting multiple cytokines or metabolites and inhibiting tumor-infiltrating CD8^+^ T cell accumulation [24]. In the current research, the effect of MPP7 on immune infiltration was assessed. The results obtained using various immune deconvolution approaches revealed that MPP7 was significantly related to immune cell infiltration, including cancer-related fibroblasts and CD8^+^ T cells, in certain tumors. Overall, our study suggests that MPP7 has the potential to be an effective target for immunotherapy to improve the health of cancer patients. However, further preclinical and clinical experiments are required to identify the connection between immune checkpoints and MPP7 expression.

MPP7 plays a significant role in tumorigenesis, but its possible mechanisms are still unclear. Functional enrichment analysis of *MPP7* co-expressed genes revealed that MPP7 may affect a variety of cancers through the Rap1 signaling pathway. The Rap1 signaling pathway plays crucial roles in multiple aspects of tumor biology, including survival, cell proliferation, migration, invasion, and metastasis [25]^.^ Nevertheless, the potential correlation between MPP7 and the Rap1 signaling pathway and the possibility of MPP7 as a cancer therapeutic target warrant further investigation.

## 5 Conclusions

This research examined the potential prognostic value, expression, genetic mutations, immunomodulatory effects, protein phosphorylation, and relevant signaling of MPP7 in various cancers using comprehensive bioinformatics analysis methods. Notably, MPP7 could be a possible prognostic and immune-related biomarker in cancer patients. Our research clarified the function of MPP7 in tumorigenesis from various perspectives, which provides a foundation for subsequent study on the specific mechanisms of MPP7 in the development and treatment of cancers.

## Availability of data and materials

Total data used to generate the consequences displayed in the research could be gained from the corresponding author upon rationale request.

## Consent for publication

All authors agree to publish the research.

## Ethics approval and consent to participate

Not applicable.

## Funding

This research was supported by grants from the Medical Science and Technology Joint Project of Henan Province (LHGJ20220745) and Open Project of the Provincial Scientific Research Platform of Henan Province Children's Hospital (SS202201).

## CRediT authorship contribution statement

**Xiaotong Xu:** Methodology. **Weyland Cheng:** Methodology. **Shuai Zhao:** Methodology. **Yuchun Liu:** Investigation. **Lifeng Li:** Investigation. **Xiaorui Song:** Investigation. **Yaodong Zhang:** Conceptualization. **Cong Ding:** Conceptualization.

## Declaration of competing interest

The authors declare that they have no known competing financial interests or personal relationships that could have appeared to influence the work reported in this paper.

## Abbreviations

ACC: Adrenocortical carcinoma
BLCA: Bladder urothelial carcinoma
BRCA: Breast invasive carcinoma
CDC50B: Cell cycle control protein 50B
CDS1: CDP-diglyceride synthase 1
COAD: Colon adenocarcinoma
DFS: Disease-free survival
DLBC: Lymphoid neoplasm diffuse large B-cell lymphoma
ESCA: Esophageal carcinoma
FBXO34: F-box only protein 34
GBM: Glioblastoma multiforme
GO: Gene Ontology
GTEx: Genotype-Tissue Expression project
HNSC: Head and neck squamous cell carcinoma
KEGG: Kyoto Encyclopedia of Genes and Genomes
KIRC: Kidney renal clear cell carcinoma
KIRP: Kidney renal papillary cell carcinoma
LAML: Acute myeloid leukemia
LGG: Brain lower grade glioma
LIHC: Liver hepatocellular carcinoma
LUAD: Lung adenocarcinoma
LUSC: Lung squamous cell carcinoma
MARVELD2: MARVEL domain-containing protein 2
MPP7: MAGUK p55 subfamily member 7
MSI: Microsatellite instability
OS: Overall survival
PAAD: Pancreatic adenocarcinoma
PRAD: Prostate adenocarcinoma
READ: Rectum adenocarcinoma
SKT: Sickle tail protein homolog
SKCM: Skin cutaneous melanoma
STAD: Stomach adenocarcinoma
TCGA: The Cancer Genome Atlas
TGCT: Testicular germ cell tumors
THCA: Thyroid carcinoma
THYM: Thymoma
TMB: Tumor mutation burden
UCEC: Uterine corpus endometrial carcinoma
UCS: Uterine carcinosarcoma
UVM: Uveal melanoma
